# Roles of immunosuppressive myeloid states in colorectal cancer checkpoint inhibitor non-response: single-cell and spatial proteomics, and reprogramming approaches

**DOI:** 10.3389/fimmu.2025.1742654

**Published:** 2026-01-06

**Authors:** Yu Dai, Xinyu Luo, Li Zhang, Jin Yan

**Affiliations:** 1 Department of Oncology, The Affiliated Hospital of Southwest Medical University, Luzhou, Sichuan, China; 2 School of Medicine, University of Electronic Science and Technology of China, Sichuan Cancer Hospital, Chengdu, Sichuan, China; 3 Department of Abdominal Medical Oncology, Sichuan Cancer Hospital & Institute, Sichuan Cancer Center, Cancer Hospital Affiliated to University of Electronic Science and Technology of China, Chengdu, Sichuan, China

**Keywords:** cancer-associated fibroblasts, checkpoint resistance, colorectal cancer, immune checkpoint inhibitors, neutrophils, tumor-associated macrophages

## Abstract

Colorectal cancer (CRC) exhibits a striking dichotomy in response to immune checkpoint inhibitors (ICIs), with durable benefit largely restricted to dMMR/MSI-H disease while most pMMR/MSS tumors remain refractory. Central to this resistance is an immunosuppressive myeloid ecosystem—dominated by SPP1high tumor-associated macrophages, neutrophils/PMN-MDSCs, and LAMP3^+^ mregDCs—that enforces chemokine-driven exclusion, attenuates antigen presentation, and sustains metabolite-mediated T-cell suppression. Despite advances in checkpoint blockade and biomarker stratification, these microenvironmental circuits constitute a major therapeutic hurdle. Moreover, single-cell and spatial proteomic platforms (e.g., CITE-seq, CODEX, imaging mass cytometry) now resolve the composition and topology of suppressive neighborhoods and highlight their utility for patient stratification and pharmacodynamic monitoring. This mini review summarizes cellular and spatial mechanisms by which myeloid states drive ICI non-response in CRC, emphasizing stromal TGF-β–coupled SPP1+ TAM programs, granulocytic chemokine axes (CXCL1/2–CXCR2; IL-8–CXCR1/2), and mregDC-mediated co-inhibition. We outline current and emerging myeloid-reprogramming strategies—including PI3Kγ and CSF1–CSF1R targeting, TREM2 antagonism, COX-2–PGE2 blockade, and adenosine-axis inhibition—and their integration with PD-(L)1 therapy, alongside single-cell/spatial endpoints to quantify on-treatment remodeling. The purpose of this mini-review is to provide a mechanistic and technology-informed framework to reference rational trial design and clinical translation for overcoming checkpoint resistance in CRC.

## 1 Introduction

Colorectal cancer (CRC) illustrates a marked dichotomy in responsiveness to immune checkpoint inhibitors (ICIs): durable clinical benefit is largely confined to the mismatch repair–deficient/microsatellite instability–high (dMMR/MSI-H) subset, whereas the majority of mismatch repair–proficient/microsatellite-stable (pMMR/MSS) tumors fail to respond (1–3). This disparity reflects fundamental differences in tumor–immune ecology and has motivated concerted efforts to define tractable barriers to T-cell–directed immunotherapy in CRC (4–6). In parallel, molecular taxonomies such as the consensus molecular subtypes (CMS) emphasize that mesenchymal, TGF-β–rich stromal programs (e.g., CMS4) coexist with immune-inflamed (CMS1) states (7–9), reinforcing the premise that context-specific microenvironmental circuits shape antitumor immunity and therapeutic outcome.

A convergent body of evidence implicates immunosuppressive myeloid populations—tumor-associated macrophages (TAMs), myeloid-derived suppressor cells (MDSCs), neutrophils, and mature dendritic cells enriched in immunoregulatory molecules (mregDCs)—as dominant effectors of checkpoint inhibitor non-response (10–12). These lineages constrain priming and execution of cytotoxic T-cell responses through impaired antigen presentation, nitric-oxide/reactive oxygen species programs, checkpoint ligand upregulation, and tolerogenic cytokine/eicosanoid/adenosine networks. Single-cell atlases in CRC further resolve functionally distinct myeloid states; for example, SPP1high macrophages that interact with fibroblast activation protein (FAP)+ cancer-associated fibroblasts and align with desmoplastic, T-cell–excluded niches (13, 14). Spatially resolved proteomic imaging corroborates that these myeloid programs assemble into reproducible “cellular neighborhoods”—recurrent, analytically defined arrangements of cell types within fixed spatial radii—at the invasive front that stratify antitumor immunity and prognosis (15–17).

Non-response phenotypes also track with granulocytic axes. Across tumor types, elevated systemic and intratumoral IL-8 (CXCL8) associates with reduced benefit from PD-(L)1 blockade, consistent with neutrophil/MDSC-mediated suppression (18, 19). In CRC, clinicopathologic inflammation and neutrophil enrichment correlate with resistance even among dMMR/MSI-H cases, underscoring that myeloid circuits can override high neoantigen load. These observations place chemokine pathways such as CXCL1/2–CXCR2, CCL2–CCR2, and IL-8–CXCR1/2—and their downstream effector programs—at the center of immune exclusion in CRC.

Methodological advances now enable rigorous deconvolution of myeloid heterogeneity and its spatial organization in human CRC. High-parameter single-cell RNA sequencing coupled to protein measurements has delineated TAM, dendritic, and neutrophil states linked to therapeutic sensitivity and resistance, and has provided mechanistic readouts for experimental perturbations (20, 21). Spatial proteomics platforms—including co-detection by indexing (CODEX) and imaging mass cytometry (IMC)—quantify dozens of proteins per cell *in situ*, allowing direct mapping of immune–stromal interfaces (22, 23), ligand–receptor topologies, and suppressive niches at single-cell resolution.

These insights nominate myeloid reprogramming as a rational complement to ICIs in CRC. Targetable nodes include PI3Kγ, a macrophage-intrinsic signaling switch whose inhibition restores inflammatory antigen presentation and augments checkpoint blockade; the CSF1–CSF1R axis that maintains immunosuppressive TAM pools; TREM2, a myeloid checkpoint whose blockade reshapes TAM states and synergizes with PD-1 therapy; and metabolic circuits such as COX-2–PGE_2_ and adenosine (CD39/CD73–A_2_A/A_2_B) that tonically enforce T-cell suppression. This review synthesizes current understanding of immunosuppressive myeloid states in CRC non-response to ICIs, with two aims: first, to integrate single-cell and spatial proteomic evidence that defines the composition and architecture of suppressive niches in human tumors; and second, to evaluate reprogramming strategies that target these myeloid circuits to convert immune-excluded CRC into ICI-responsive disease. The intent is to provide a mechanistic and technology-informed framework to guide study design and clinical translation in CRC immunotherapy.

## 2 Landscape of immunosuppressive myeloid states in colorectal cancer

**Figure 1:**
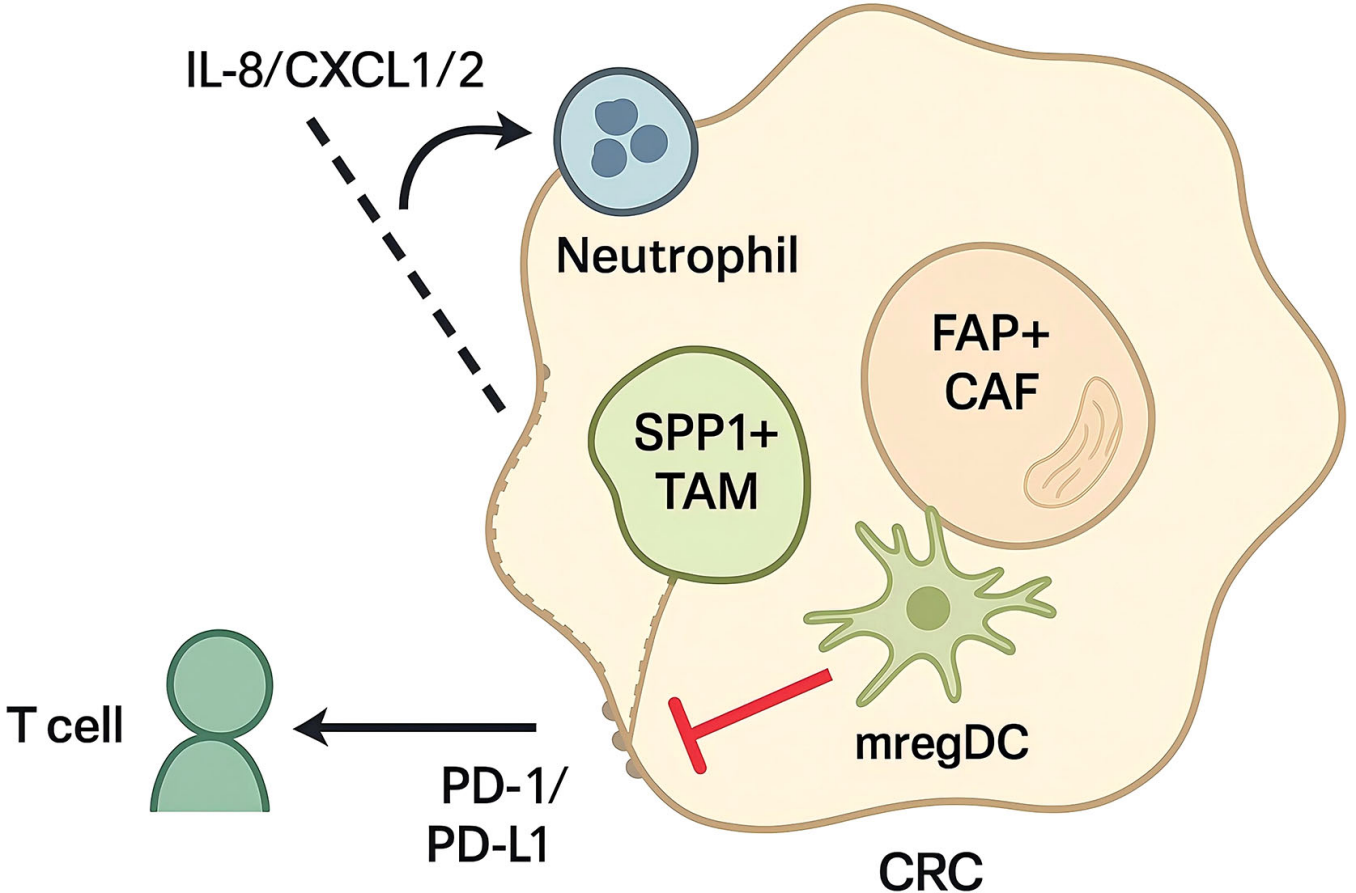
Immunosuppressive myeloid–stromal crosstalk underlying PD-1/PD-L1 resistance in the CRC tumor microenvironment.

Across colorectal tumors, the immunosuppressive myeloid compartment comprises lineage-defined populations—tumor-associated macrophages, neutrophils/PMN-MDSCs, monocytic-MDSCs, and dendritic cells—and finer “states” defined by transcriptional and protein markers shared across these cells. As shown in **Figure 1**, single-cell profiling in human CRC delineates macrophage states that diverge from the canonical M1/M2 schema; among these, SPP1high macrophages are consistently expanded and associate with extracellular matrix remodeling, VEGF/EGF signaling, and chemokine outputs that favor T-cell exclusion (24, 25). While transcriptomic data indicate that these cells are typically enriched for immunoregulatory transcripts such as TREM2, ARG1, IL10 and PD-L1. Within this framework, SPP1high macrophages—predominantly monocyte-derived and overlapping with but not identical to classical M2-like TAMs—are consistently expanded, with evidence for angiogenic versus matrix-remodeling SPP1^+^ subclusters that mirror analogous populations in other gastrointestinal malignancies. Spatial analyses demonstrate that SPP1high macrophages colocalize with fibroblast activation protein (FAP)+ cancer-associated fibroblasts at the invasive front, forming a mesenchymal–myeloid circuit linked to desmoplasia, poor cytotoxic infiltration, and adverse outcome (26, 27). These observations situate SPP1high macrophages as a central suppressive node in CRC.

Granulocytic axes are prominent in CRC immune evasion. Neutrophils and PMN-MDSCs accumulate through CXCL1/2–CXCR2 and IL-8/CXCR1/2 signaling, amplify reactive oxygen species and arginase programs, and enforce immune exclusion. Pharmacologic and genetic perturbations across gastrointestinal and colitis-associated models demonstrate that CXCR2 signaling governs granulocytic recruitment and tumor promotion, providing mechanistic support for therapeutic targeting of this pathway (28, 29). Clinically, elevated circulating IL-8 tracks with inferior outcomes to PD-(L)1 blockade across malignancies, consistent with a neutrophil-dominated suppressive milieu that can supersede high tumor antigenicity (30, 31). In CRC, emergent data nominate combined CCR1/CXCR2 blockade to curtail protumor myeloid trafficking, underscoring the therapeutic relevance of chemokine circuits (32, 33).

Dendritic cell lineages in CRC encompass conventional DC1/2 and a mature LAMP3^+^ state enriched in immunoregulatory molecules (mregDC) that upregulates PD-L1, PD-L2 and other tolerogenic mediators while retaining antigen-presentation machinery (34, 35). This program, induced upon tumor-antigen uptake, supports T-cell priming in a context that simultaneously delivers checkpoint and cytokine-based inhibitory signals, thereby tuning effector differentiation and exhaustion. These DC states participate in structured cellular neighborhoods identified by multiplexed imaging; CRC tissues harbor myeloid-attracting hubs at the tumor–luminal interface and MMRd-enriched immune hubs within tumor nests (36, 37), each with characteristic myeloid–lymphoid assemblies that stratify prognosis and therapeutic sensitivity.

CRC harbors a reproducible landscape of suppressive myeloid states anchored by SPP1high tumor-associated macrophages, CXCR2-driven granulocytic programs, and mregDC modules. Single-cell and spatial studies converge on the concept that these populations assemble into mesenchymal-skewed, chemokine-rich niches that impede T-cell recruitment and function (38, 39). These features provide mechanistic context for checkpoint inhibitor non-response in pMMR/MSS disease.

## 3 Single-cell and spatial proteomics for mapping suppressive niches

Single-cell multi-omic profiling and high-plex spatial proteomics provide complementary resolution on how immunosuppressive myeloid programs are organized in colorectal cancer (40, 41). Antibody-tagged single-cell transcriptomics (CITE-seq, REAP-seq) jointly quantifies transcripts and surface proteins, enabling direct measurement of checkpoint ligands, antigen-presentation molecules, chemokine receptors, and myeloid differentiation markers within the same cells and thereby resolving SPP1^high^ TAMs, mregDCs, and granulocytic states with reduced label ambiguity (42, 43). As shown in **Table 1**, these platforms define intracellular programs, whereas high-parameter imaging modalities capture the topologies that enforce T-cell exclusion. CITE-seq and REAP-seq are established for simultaneous RNA–protein readouts and support robust annotation of myeloid states that are otherwise weakly separable by mRNA alone.

**Table 1:** Single-cell and spatial proteomic platforms for delineating immunosuppressive niches in colorectal cancer.

Platform	Modality/readout	Specimen & scale	Typical utility for suppressive-niche mapping	Notes
CITE-seq/REAP-seq	Droplet scRNA-seq with antibody-derived tags (RNA+protein)	Fresh/viable single-cell suspensions; ≥10^4^ cells	Define TAM, mregDC, MDSC, neutrophil states; quantify PD-L1, HLA-DR, CSF1R, CXCR2, AXL at single-cell level	Panel validation; isotype/background controls; batch correction and doublet removal
CODEX	DNA-barcoded antibody cyclic IF (≥40–60 proteins)	FFPE sections; whole fields or tissue microarrays	Map cellular neighborhoods at the invasive front; measure myeloid–CAF–T-cell interfaces and checkpoint ligand densities	Antibody conjugation/FFPE reactivity; spillover correction; segmentation QC
Imaging Mass Cytometry (Hyperion IMC)	Metal-tagged antibody TOF imaging (≈30–40 proteins)	FFPE/Frozen; tiles to whole regions	Quantify cell–cell proximities and gradients; identify neutrophil/MDSC corridors and macrophage rings	Metal channel optimization; cross-slide normalization; proximity/ripley statistics
MIBI-TOF	Secondary-ion mass spectrometry of metal-conjugated antibodies	FFPE; high-resolution fields	Stable quantification across batches; fine mapping of ligand–receptor borders	Vacuum stability, depth profiling; rigorous panel calibration

CITE, cellular indexing of transcriptomes and epitopes; REAP, RNA end-associated purification; TAM, tumor-associated macrophage; mregDC, mature dendritic cells enriched in immunoregulatory molecule; MDSC, myeloid-derived suppressor cell; CODEX, co-detection by indexing; IF, immunofluorescence; FFPE, formalin-fixed paraffin-embedding; CAF, cancer-associated fibroblast; QC, quality control; IMC, imaging mass cytometry; MIBI-TOF, multiplexed ion beam imaging by time of flight.

Multiplexed spatial imaging has established that CRC assembles reproducible “cellular neighborhoods” at the invasive front in which myeloid, stromal, and lymphoid elements co-cluster. Using 56-marker CODEX on CRC tissue microarrays, Some studies identified coordinated neighborhoods whose composition stratified immune pressure and outcome, directly implicating organized myeloid–stromal hubs in immune control (44–46). Spatial multi-omic analyses consistently show tight coupling of SPP1^+^ macrophages with FAP^+^ cancer-associated fibroblasts, a mesenchymal–myeloid circuit linked to extracellular-matrix remodeling and T-cell exclusion in independent CRC cohorts and validated by spatial transcriptomics and immunofluorescence (47, 48). Three-dimensional high-plex atlases further reveal gradients and barriers not apparent in two dimensions, quantify cell–cell distances (49, 50), and uncover nested suppressive structures that align with histologic fronts and glandular architecture in CRC.

These spatial readouts contextualize mechanistic nodes relevant to non-response. Lymphatic-adjacent Treg–mregDC niches form immunoregulatory hubs that restrict antigen access and can be quantified by CODEX/IMC to nominate actionable checkpoints and cytokine axes. Spatially mapped TGF-β activity aligns with induction of immunosuppressive SPP1^+^ macrophages and impaired T-cell recruitment, reinforcing a dual stromal–myeloid barrier that is measurable with multiplexed imaging and suitable for pharmacodynamic monitoring in trials that combine myeloid-reprogramming agents with PD-(L)1 blockade.

## 4 Mechanistic circuits connecting myeloid programs to checkpoint inhibitor failure

CRC non-response to ICIs maps to stromal–myeloid circuits that restrict T-cell access, blunt priming, and attenuate effector function. Single-cell and spatial analyses position SPP1high tumor-associated macrophages in tight contact with FAP+ cancer-associated fibroblasts at the invasive front, enriched for extracellular-matrix and TGF-β signaling; this configuration aligns with immune-excluded architecture and adverse outcome (51, 52). Functional inference and experimental data support SPP1–CD44 and TGF-β–SMAD2/3/4 programs, coupled to PI3Kγ–AKT signaling and integrated with CSF1, CXCL12, CCL2, VEGF, and complement components, which stabilize SPP1^+^/TREM2^+^ macrophage identity, skew antigen presentation, and exclude cytotoxic lymphocytes (53, 54). In immune-excluded tumors, stromal TGF-β blockade restores intratumoral T-cell penetration and sensitizes to PD-(L)1 therapy, nominating this mesenchymal–myeloid axis as a proximal cause of checkpoint failure.

Chemokine pathways couple granulocytic recruitment to resistance. CXCL1/2–CXCR2 and IL-8/CXCR1/2 signaling drive accumulation of neutrophils and PMN-MDSCs that generate reactive oxygen species and arginase programs, degrade chemokine gradients, and physically occlude T-cell entry (55, 56). Clinically, elevated baseline or on-treatment IL-8 tracks with reduced benefit from PD-(L)1 blockade across solid tumors, supporting IL-8/CXCR2 as a resistance biomarker and therapeutic target; CXCR2 inhibition increases effector T-cell infiltration and potentiates immunotherapy in preclinical models (57, 58).

Antigen handling and co-inhibitory signaling by dendritic cells constitute a second node linking myeloid states to non-response. Mature, migratory LAMP3^+^ dendritic cells (mregDCs) upregulate PD-L1/PD-L2 and immunoregulatory cytokines while preserving antigen-presentation machinery (59, 60); in tumors, these cells accumulate at lymphoid–stromal interfaces where they support constrained priming and program T-cell exhaustion under high checkpoint ligand density.

Macrophage-intrinsic signaling and metabolic circuits sustain these barriers and directly antagonize PD-1–directed reinvigoration. PI3Kγ signaling in macrophages enforces a C/EBPβ-driven program that suppresses antigen presentation and promotes IL-10/ARG1 output; pharmacologic PI3Kγ inhibition reprograms TAMs and synergizes with PD-1 blockade. TREM2+ lipid-associated TAMs curtail T-cell function, and anti-TREM2 augments PD-1 therapy in resistant settings (61, 62). COX-2–PGE_2_ signaling and PMN-MDSC-intrinsic FATP2-dependent arachidonic-acid metabolism amplify PGE_2_ production, reinforce myeloid suppression, and reduce responsiveness to ICIs; early clinical experience indicates feasibility of combining COX inhibition with PD-1 in MSI-H CRC (63, 64). Adenosine produced through CD39/CD73 engages A_2_A/A_2_B receptors on myeloid and lymphoid cells to blunt effector responses, implicating ectonucleotidases and adenosine-receptor antagonists as complementary approaches (65, 66). In CRC models, CSF1R inhibition reprograms TAMs and potentiates anti-PD-1 efficacy, further highlighting the tractability of these circuits (67, 68).

These pathways converge on T-cell signal deprivation and signaling interference: ARG1-mediated L-arginine depletion downregulates CD3ζ and arrests T-cell cycling, while iNOS-derived nitric oxide and peroxynitrite from MDSCs nitrates T-cell receptors and impairs antigen recognition, consolidating myeloid control over effector competence in CRC.

## 5 Reprogramming strategies targeting myeloid compartments

Therapeutic reprogramming of myeloid lineages in CRC aims to restore antigen presentation, diminish suppressive cytokine–eicosanoid–adenosine signaling, and reduce chemokine-driven exclusion of effector T cells. Macrophage-intrinsic phosphoinositide 3-kinase-γ (PI3Kγ) functions as a signaling switch that biases tumor-associated macrophages (TAMs) toward immunosuppression; pharmacologic PI3Kγ inhibition rebalances NF-κB/C/EBPβ programs, enhances antigen-presenting functions, and potentiates PD-(L)1 blockade in preclinical models, supporting clinical evaluation as a combinatorial partner (69, 70). Complementarily, targeting the CSF1–CSF1R axis reduces and re-educates suppressive TAM pools and increases intratumoral CD8+ T-cell infiltration. In CRC models, CSF1R inhibition augmented anti–PD-1 efficacy, indicating on-target remodeling of macrophage phenotypes (71, 72). However, CSF1R blockade can induce compensatory granulocytic recruitment through cancer-associated fibroblast chemokines, arguing for rational pairing with chemokine-axis inhibitors or T cell–priming agents to prevent myeloid rebound.

TREM2 defines an immunosuppressive TAM state that accumulates across solid tumors, including colorectal contexts. Genetic ablation or antibody blockade of TREM2 reorganizes the myeloid compartment toward inflammatory phenotypes, increases intratumoral T-cell activity, and synergizes with PD-1 blockade *in vivo* (73, 74). Mechanistic and translational studies further support TREM2 as a therapeutically tractable macrophage checkpoint; effector-enhanced anti-TREM2 antibodies and modalities delivering TREM2-directed binders into the tumor bed improve responses to PD-1 therapy, including in CRC models (75, 76). These position TREM2 antagonism as a candidate backbone for myeloid reprogramming in checkpoint-refractory CRC.

Granulocytic programs that enforce immune exclusion can be attenuated by interrupting chemokine-driven trafficking. In CRC, preclinical and early translational evidence supports blockade of IL-8/CXCL1/2–CXCR1/2 pathways to reduce neutrophil/PMN-MDSC accumulation and improve sensitivity to immune checkpoint blockade. Dual inhibition of CCR1 and CXCR2 curtailed myeloid recruitment and exhibited antitumor activity in colorectal settings, providing a strategy to counteract chemokine redundancy and stromal compensation observed with single-node targeting (77–80). Broader analyses of CXCR1/2 therapeutics corroborate that neutrophil-directed inhibition can be integrated with ICIs where granulocytic exclusion dominates (81, 82).

Metabolic and purinergic circuits that stabilize suppressive myeloid states are tractable points of intervention. COX-2–PGE_2_ signaling dampens dendritic-cell function, promotes regulatory myeloid outputs, and constrains T-cell priming; selective EP2/EP4 pathway inhibition or COX blockade reverses these effects in models, and early clinical experience combining COX inhibition with PD-1 in dMMR metastatic CRC indicates feasibility and activity, motivating expansion into pMMR disease (83, 84). In PMN-MDSCs, fatty-acid transport protein-2 (FATP2/SLC27A2) drives arachidonic-acid uptake and PGE_2_ synthesis; FATP2 inhibition abrogates granulocytic suppression and enhances checkpoint responses *in vivo*, nominating a selective strategy to disable neutrophil-dominant resistance (85, 86). The adenosinergic axis (CD39/CD73–A_2_A/A_2_B) tonically suppresses myeloid and lymphoid effector functions; co-blockade of CD73 and A_2_A receptors increases antitumor immunity and synergizes with ICIs in preclinical systems, and multiple agents targeting this pathway are under clinical evaluation (87, 88). Because macrophages, neutrophils, and MDSCs are also crucial for host defense and tissue repair, myeloid-targeted combinations may increase infection risk, delay wound healing, and trigger on-target inflammatory toxicities that must be carefully profiled in CRC trials.

## 6 Conclusion and outlook

Across colorectal cancer, convergent single-cell and spatial proteomic evidence identifies immunosuppressive myeloid programs as primary constraints on checkpoint inhibitor efficacy. SPP1high macrophages coupled to fibroblast-rich, TGF-β–dominant stroma, CXCR2-driven granulocytic circuits, and LAMP3^+^ mregDC modules form interconnected barriers that limit T-cell priming, trafficking, and effector function (89, 90). These populations assemble into reproducible cellular neighborhoods—particularly at the invasive front—where ligand–receptor density, matrix architecture, and chemokine gradients align to produce T-cell exclusion and checkpoint non-response. This integrated model explains why pMMR/MSS tumors rarely benefit from PD-(L)1 blockade and why even dMMR/MSI-H disease can be overridden by dominant myeloid axes.

Methodological advances now enable precise dissection of these states and their spatial context in clinical specimens. Joint RNA–protein single-cell profiling distinguishes closely related myeloid phenotypes and quantifies actionable surface molecules, while high-plex imaging resolves cell–cell proximities, gradients, and compartmentalized signaling. As shown in **Table 1**, these platforms provide complementary views of composition and topology and are sufficiently mature for biomarker development and pharmacodynamic assessment. Standardized panel design, segmentation quality control, and cross-batch normalization remain essential to ensure reproducibility across centers and trial cohorts.

Therapeutically, reprogramming—not ablation—of myeloid compartments emerges as the most generalizable path to synergy with PD-(L)1 blockade. Macrophage-intrinsic switches (PI3Kγ, TREM2), lineage-support pathways (CSF1–CSF1R), neutrophil trafficking axes (IL-8/CXCL1/2–CXCR1/2; CCR1/CCR2), and immunometabolic circuits (COX-2–PGE_2_; FATP2-dependent lipid handling; CD39/CD73–A_2_A/A_2_B adenosine signaling) constitute tractable nodes that can be combined to dismantle redundant suppressive loops. The preclinical and early translational signal across these targets supports a development strategy that prioritizes rational doublets with PD-(L)1 and favors mechanisms that simultaneously improve antigen presentation, restore chemokine balance, and reduce metabolite-mediated T-cell suppression (91, 92). Because pathway compensation is common, combinations that pair macrophage re-education with neutrophil-axis interruption, or that couple stromal TGF-β modulation with myeloid-targeted agents, are likely to be required in immune-excluded CRC.

Translation will depend on trial designs that embed single-cell and spatial endpoints. Neoadjuvant “window” studies and trials in metastatic CRC should use on-treatment biopsies to quantify state shifts in SPP1high TAMs, neutrophil/MDSC densities, mregDC prevalence, checkpoint-ligand distribution, and T-cell access to tumor nests. Peripheral biomarkers—such as circulating IL-8 and blood-derived myeloid signatures—can provide complementary, low-burden monitoring but should be analytically bridged to tissue-resolved readouts. Prospective stratification by stromal–myeloid architecture (e.g., mesenchymal/TGF-β–high niches) and by chemokine-axis activity will reduce biological heterogeneity and increase the likelihood of detecting true drug–drug interactions.

Looking forward, three priorities are clear. First, harmonized taxonomies of CRC myeloid states, grounded in cross-platform reference atlases, are needed to stabilize target selection and endpoint interpretation. Second, pharmacodynamic standards that tie quantitative remodeling of suppressive neighborhoods to clinical benefit should be codified and used to advance only those combinations that meet predefined tissue-based thresholds. Third, iterative co-development of drugs and diagnostics—integrating single-cell, spatial, and circulating measures—will be essential to match specific myeloid barriers with the most appropriate reprogramming strategies. By aligning mechanism, measurement, and patient selection, it should be possible to convert the immune-excluded colorectal tumor into a context receptive to checkpoint reinvigoration and to broaden durable benefit beyond the dMMR/MSI-H subset.
